# The Role of Androgen Receptor Signaling in Ovarian Cancer

**DOI:** 10.3390/cells8020176

**Published:** 2019-02-19

**Authors:** Taichi Mizushima, Hiroshi Miyamoto

**Affiliations:** 1 Department of Pathology & Laboratory Medicine, University of Rochester Medical Center, Rochester, NY 14642, USA; mizu123shima@gmail.com; 2 James P. Wilmot Cancer Institute, University of Rochester Medical Center, Rochester, NY 14642, USA; 3 Department of Obstetrics and Gynecology, Yokohama City University Graduate School of Medicine, Yokohama 236-0004, Japan; 4 Department of Urology, University of Rochester Medical Center, Rochester, NY 14642, USA

**Keywords:** androgen, androgen receptor, carcinogenesis, ovary, tumor progression

## Abstract

Emerging evidence has suggested that androgen receptor signaling plays an important role in ovarian cancer outgrowth. Specifically, androgen receptor activation appears to be associated with increased risks of developing ovarian cancer and inducing tumor progression. However, conflicting findings have also been reported. This review summarizes and discusses the available data indicating the involvement of androgens as well as androgen receptor and related signals in ovarian carcinogenesis and cancer growth. Although the underlying molecular mechanisms for androgen receptor functions in ovarian cancer remain far from being fully understood, current observations may offer effective chemopreventive and therapeutic approaches, via modulation of androgen receptor activity, against ovarian cancer. Indeed, several clinical trials have been conducted to determine the efficacy of androgen deprivation therapy in patients with ovarian cancer.

## 1. Introduction

Ovarian cancer comprising a variety of histological subtypes is one of the most common types of gynecological malignancy, with an estimate of 295,414 new cases and 184,799 deaths occurring in 2018 worldwide [1]. It is often diagnosed at an advanced stage, presumably due to its asymptomatic characteristics, and still represents a deadly disease despite significant advances in treatment strategies. Further investigation is thus required to develop an understanding of the pathogenesis and pathophysiology of ovarian cancer. This may consequently provide novel targeted therapy to improve patient outcomes.

The biological actions of androgens, including testosterone and dihydrotestosterone as well as those primarily derived from the adrenal gland (and ovary) (e.g., dehydroepiandrosterone (DHEA) and its sulfated form (DHEA-S), androstenedione, androstenediol)), are normally mediated through the androgen receptor (AR), a ligand-inducible transcription factor belonging to the steroid hormone receptor superfamily [2,3]. The diverse functions of androgen-mediated AR are known even in females and include hair growth, libido, muscle strength and volume, erythropoietin production, bone formation/growth, and differentiation/maturation of bone marrow stem cells. AR signaling has also been implicated in the pathogenesis and growth of malignancies, including not only prostate cancer for which androgen deprivation therapy remains the mainstay of management [2] but also other types such as breast cancer [4] and bladder cancer [3]. In particular, similar to the observations in prostate cancer, preclinical studies have demonstrated that activation of AR-related signals is generally associated with induction of urothelial tumorigenesis and tumor progression [3].

Previous studies, using AR knockout mice, indicated physiological functions of AR in the ovary [5,6,7]. Specifically, loss of AR in granulosa cells resulted in impairment of their differentiation and follicle growth/maturation. Meanwhile, an increasing amount of evidence has indicated the involvement of AR and related signals in the development and progression of ovarian cancer. In this article, we review available data suggesting their modulation through the AR pathway and discuss underlying molecular mechanisms.

## 2. Role of Androgens and AR Signaling in Ovarian Carcinogenesis

A variety of factors are known to involve the modulation of AR transcriptional activity. These include not only various androgens and other androgenic compounds but also the expression levels and variants of AR itself. In this section, we describe epidemiological/clinical and preclinical observations indicating potential involvement of AR signaling in ovarian tumorigenesis.

### 2.1. Androgen Levels and Ovarian Cancer Risk

A prospective study published in 1985 assessed the urinary concentrations of DHEA as well as metabolites of testosterone, androsterone, and etiocholanolone, in 1484 women, 12 of whom subsequently developed ovarian cancer and found that the levels of DHEA (*P* = 0.007), androsterone (*P* = 0.06), and etiocholanolone (*P* = 0.33) in ovarian cancer patients were lower than those in control subjects [8]. Another prospective study published in 1995 compared the serum levels of adrenal androgens in 31 patients with ovarian cancer versus 62 control women matched on race, age, and menopausal status, as well as the number of days from the beginning of the last menstrual period (in premenopausal women) and year from the last menstrual period (in postmenopausal women) [9]. It found that the levels of DHEA (15.9 ± 13.2 versus 9.7 ± 6.6 nmol/L; *P* = 0.02) and androstenedione (4.5 ± 2.8 versus 3.3 ± 2.1 nmol/L; *P* = 0.03) were significantly higher in cancer patients than in control subjects, but the difference in those of DHEA-S (4.1 ± 1.4 versus 3.2 ± 2.2 µmol/L; *P* = 0.21) was not statistically significant between the two groups. In particular, the odds ratio (OR) for the risk of ovarian cancer in those with high androstenedione was 7.6 (95% confidence interval (CI) = 1.2–48.7; *P* = 0.008), compared to those with low androstenedione. In addition, considerable differences in the levels of DHEA (23.9 ± 15.6 versus 11.4 ± 5.9 nmol/L; *P* = 0.02) and androstenedione (4.9 ± 2.8 versus 3.4 ± 1.7 nmol/L; *P* = 0.05) were still observed in premenopausal women (n = 13), but not in postmenopausal women (n = 18). By contrast, subsequent studies [10,11,12,13] failed to show significant differences in the blood levels of DHEA, DHEA-S, and/or androstenedione between all ovarian cancer patients (n = 132–565 in each study) versus matched controls (n = 258–1097). However, subgroup analyses further showed an inverse association between the level of DHEA and the risk of ovarian cancer in postmenopausal women (relative risk = 0.65, 95% CI = 0.36–1.19, *P* = 0.04) [12] as well as significant associations between the levels of DHEA-S and the development of serous tumors (OR = 0.89), “type I” tumors (e.g., low-grade serous/endometrioid/mucinous tumors, Brenner tumor) (OR = 1.41), or “type II” tumors (e.g., high-grade serous/endometrioid carcinomas, malignant mixed Müllerian tumor, undifferentiated carcinoma) (OR = 0.86), and between the levels of androstenedione and the development of serous tumors (OR = 0.79), low grade tumors (OR = 1.99), high grade tumors (OR = 0.75), type I tumors (OR = 1.99), or type II tumors (OR = 0.71) [13].

The blood concentrations of testosterone have also been assessed for the risk of ovarian cancer. Although the studies described above [10,11,12,13] have demonstrated no strong associations of the levels of testosterone and free testosterone with overall incidence of ovarian cancer, free testosterone concentrations were found to inversely associate with ovarian cancer risk in postmenopausal women (OR = 0.45, 95% CI = 0.24–0.86, *P* = 0.01) [11] or serous tumor risk (OR = 0.90, 95% CI = 0.75–1.08, *P* = 0.02) [13].

These findings suggest the involvement of androgens in modulating the development of ovarian cancer. Of note, serum concentrations of androgens have been shown not to always correlate with their follicular fluid concentrations, presumably due to the avascularity of the ovarian surface epithelium [14,15]. Accordingly, it is suggested that, compared with circulating hormone levels, androgens from ovarian origin, especially in premenopausal women, play a more important role in the pathogenesis of ovarian cancer.

### 2.2. Polycystic Ovary Syndrome (PCOS) or Obesity and Ovarian Cancer Risk

PCOS as well as obesity in women of reproductive age is known to associate with elevated levels of androgens. A preliminary study involving 7 ovarian cancer patients revealed 2.5-fold higher incidence of ovarian cancer (95% CI = 1.1–5.9) in those with a history of PCOS [16]. This study further showed that the risk of ovarian cancer was even higher in those who had body mass index (BMI) of 13.3–18.5 kg/m^2^ at age 18 (OR = 15.6, 95% CI = 3.4–71.0) [16]. A larger case-control study involving 1276 cases of invasive ovarian carcinoma and 315 cases of borderline tumor conducted in Australia showed an increased risk of serous borderline tumors in those with a history of PCOS (OR = 2.5, 95% CI = 1.0–6.1) [17]. However, no strong association between the risk of invasive cancer and PCOS (OR = 0.8, 95% CI = 0.4–1.6) [17]. A recent study involving 41 (2.8%) and 37 (2.3%) PCOS patients with and without ovarian epithelial malignancy, respectively, also demonstrated no significant increase in the incidence of ovarian cancer in those with PCOS (OR = 0.97, 95% CI = 0.61–1.56) [18]. Similarly, controversial data in population-based case-control studies showing a strong association (overall OR = 1.17, 95% CI = 0.85–1.62, *P* = 0.006; OR for serous borderline tumor = 2.29, 95% CI = 1.32–3.98, *P* = 0.0008) [18] and no significant association [19] between high BMI and ovarian tumor risk have been reported. Even in the latter study [19], however, BMI was found to positively associate with cancer risk in premenopausal women. Importantly, there must be various changes, other than those of androgen levels, in women with PCOS or obesity. Thus, they are unlikely to be independent risk factors for ovarian cancer, even if androgens indeed promote ovarian tumorigenesis.

### 2.3. Oral Contraceptives or Androgenic/Anti-Androgenic Agents and Ovarian Cancer Risk

Oral contraceptives have been known to reduce androgen levels [20] at least partially via inhibiting their synthesis in the ovary. The balance between androgenic activity of the drugs and suppression of endogenous androgens may thus affect the occurrence of ovarian cancer. In the case-control Steroid Hormone and Reproduction (SHARE) Study [21], 568 ovarian cancer cases and 1026 controls were assessed to determine if oral contraceptives could alter the risk of ovarian cancer. It demonstrated that androgenic (OR = 0.52, 95% CI = 0.35–0.76) and non-androgenic (OR = 0.59, 95% CI = 0.45–0.78) oral contraceptives similarly provided significant reduction in cancer risks after the adjustment for age, number of live births, family history of ovarian cancer, and tubal ligation. There were also no significant differences in the duration of oral contraceptive use, age at first oral contraceptive use, and time since last use. These observations suggest that the androgenicity of oral contraceptives have little impact on their chemopreventive effects. Additionally, in the case-control study described above [18], the use of oral contraceptives for <5 years was shown to reduce the risk of overall ovarian tumors (OR = 0.60, 95% CI = 0.41–0.90, *P* = 0.001) or serous borderline tumors (OR = 0.49, 95% CI = 0.20–1.20, *P* = 0.0003). Nonetheless, it is noteworthy to mention that oral contraceptives alter the levels of other sex hormones, including estrogens and progesterone, which are also known to contribute to ovarian tumorigenesis [14].

In female patients, androgenic and anti-androgenic medications have been commonly used for the treatment of, for example, endometriosis. In a combination of two population-based case-control studies involving 1373 ovarian cancer patients and 1980 controls, a synthetic androgen danazol (n = 19) was found to significantly increase the incidence of ovarian cancer (OR = 3.2, 95% CI = 1.2–8.5), while the use of gonadotropin-releasing hormone agonists that ultimately reduce testosterone and estradiol, including leuprolide and nafarelin (n = 23), did not significantly affect it (OR = 1.0, 95% CI = 0.4–2.4) [22]. Similar effects of danazol (OR = 2.9, 95% CI = 1.0–8.5) or leuprolide/nafarelin (OR = 1.4, 95% CI = 0.5–4.1) were seen in a subgroup of patients with endometriosis [22]. The Australian case-control study [18] also showed an increased risk of ovarian tumors by the use of testosterone supplements (tablets, patches, troches, and cream) (n = 15, OR = 3.7, 95% CI = 1.1–12.0), but not by danazol (n = 18, OR = 1.0, 95% CI = 0.4–2.9).

### 2.4. AR Expression in Non-Neoplastic Ovary and Ovarian Cancer Tissues

*AR* gene expression has been detected via PCR-based methods in all 4 [23] and 8 [24] primary cell cultures established from human normal ovarian surface epithelium in respective studies. PCR analyses also showed the presence of the *AR* transcript in 2 of 4 primary cultures from ovarian cancer and 1 (i.e., SKOV3) of 3 established ovarian cancer cell lines [23].

Immunohistochemical studies in surgical specimens then revealed the expression of AR protein in 43% [25] and 100% [24] of human normal ovaries. In the rat ovary, localization of AR was examined: AR was positive in granulosa cells of primary and secondary follicles (strongest), surface epithelium, and theca cells of follicles and corpus luteum [26]. AR immunoreactivity was also detected in 40% of benign epithelial neoplasms and 61% of adenocarcinomas [25], as well as in 64% of ovarian neoplasms (8 serous carcinomas, 3 endometrioid carcinomas, 2 mucinous carcinomas, 1 granulosa tumor) [26], obtained from women undergoing oophorectomy. In another immunohistochemical study using surgical specimens, the levels of AR expression were significantly higher in serous carcinomas (n = 7) than in normal/inactive ovary tissues (n = 7) [27]. In addition, western blotting showed AR expression in 69% of human serous carcinoma cases [28] as well as in the OVCAR-3 human ovarian cancer cell line [26,29].

### 2.5. AR CAG Repeat Polymorphisms and Ovarian Cancer Risk

The *AR* gene contains a polymorphic CAG repeat segment coding for a polyglutamine sequence in exon 1. The length of the CAG repeats has been shown to inversely correlate with AR transcriptional activity in various types of cells, including ovarian cancer cells [30,31,32]. In primary cultures established from ovarian epithelium, CAG repeat lengths were found to be significantly shorter in cancer cells (mean: 20.6) than in normal cells (mean: 23.4) [33].

In two studies comprising patients with both hereditary and sporadic ovarian cancers, those with shorter CAG repeats were shown to be diagnosed an average of 7.2 (CAG <15; 95% CI = 2.3–12.1, *P* = 0.004) [34] or 10.45 (CAG ≤22; 95% CI = 1.28–19.62, *P* = 0.02) [35] years earlier than those with longer repeats. Correspondingly, as seen in prostate cancer [36], case-control studies involving ovarian cancer patients and unaffected control subjects have indicated that short CAG repeats are associated with a significantly higher risk of developing ovarian tumor in African Americans in the US [37], Polish Caucasians [38], or Han Chinese [39,40]. In one of these studies involving the largest number of cases (i.e., 2795 cancer patients and 2800 controls) [39], women with longer (≥22) CAG repeats were shown to be at 31% lower risk for ovarian cancer (OR = 0.69, 95% CI = 0.62–0.77, *P* = 5.06 × 10^−11^). By contrast, an increased risk of ovarian cancer was observed in women with the CAG repeats of ≥22 (OR = 2.17, 95% CI = 1.10–4.27 [41]; OR = 1.15, 95% CI = 0.92–1.43 [42]). However, other studies have demonstrated no strong associations between the length of the CAG repeats and the risk of ovarian cancer [43,44]. No significant association between the CAG repeat lengths and ovarian cancer risk in women with germline mutations of *BRCA1*, a common cause of hereditary breast-ovarian cancer syndrome, has also been reported [45]. Interestingly, BRCA1 has been suggested to function as an AR co-activator [46], although mutant BRCA1 may not be able to bind the AR [45]. These conflicting findings on the relationship between the CAG repeat length and ovarian cancer risk may be due to the diversity of absolute average repeat number in each study or androgen concentration of each case.

### 2.6. In Vitro Studies Assessing the Involvement of Androgen-Mediated AR Signaling in Ovarian Tumorigenesis

There appear to be no direct evidence from in vitro studies suggesting the involvement of androgen-mediated AR signaling in ovarian tumorigenesis. Instead, it has been shown in primary cultures established from human normal ovarian surface epithelium that testosterone and dihydrotestosterone (DHT) [47], as well as a synthetic androgen mibolerone [24], considerably induce cell proliferation. Mibolerone (100 nM) was also shown to significantly reduce apoptosis in 3 of 5 primary cell cultures examined [24]. In addition, the stimulatory effects of testosterone (10 nM) on cell proliferation of primary cultures were blocked by an AR antagonist hydroxyflutamide (10 or 100 µM) [47]. Thus, the proliferative effects of androgens might promote neoplastic transformation of ovarian epithelial cells. Meanwhile, an earlier study showed only marginal effects of DHT treatment (100 nM) for up to 10 days on the growth of a primary cell culture established from human normal ovarian surface epithelium [48].

### 2.7. In Vivo Studies Assessing the Involvement of Androgen-Mediated AR Signaling in Ovarian Tumorigenesis

Several studies have assessed the impact of androgens on ovarian tumorigenesis, using in vivo models or human patients.

In 3 of 3 guinea pigs that received testosterone, benign epithelial cysts larger than 1.5 mm (2.5 cm bilateral cysts in one of the guinea pigs), as well as papillary excrescences, were found [49]. In addition, one of these testosterone-treated guinea pigs developed large bilateral cystadenomas. These findings suggest that androgen induces the growth of ovarian surface epithelial cells. Interestingly, the level of testosterone in the wall of the ovarian cyst in guinea pigs treated with testosterone was 3-fold higher than that in their serum [49].

17β-Hydroxysteroid dehydrogenase 1 is an enzyme encoding the *HSD17B1* gene in humans and is responsible for the conversion between androstenedione and testosterone (as well as between estrone and estradiol). In adult female transgenic mouse lines universally overexpressing HSD17B1 that had elevated concentrations of testosterone (and estradiol) at embryonic day 17.5, ovarian surface epithelium hyperplasia and/or benign serous cystadenoma were found [50]. These phenotypes in the transgenic mice were then rescued by prenatal treatment with an anti-androgen flutamide or prepubertal transplantation of wild-type ovaries, suggesting their induction primarily by androgen, but not estrogen.

Additionally, cases of serous types of cancer (n = 2) [51] and endometrioid carcinoma (n = 1) [52] have been reported in female-to-male transgender patients who received testosterone supplementation. These observations in animal models and humans suggest the contribution of high androgen concentrations to inducing ovarian tumorigenesis.

## 3. Role of Androgens and AR Signaling in Ovarian Cancer Progression

**Figure 1:**
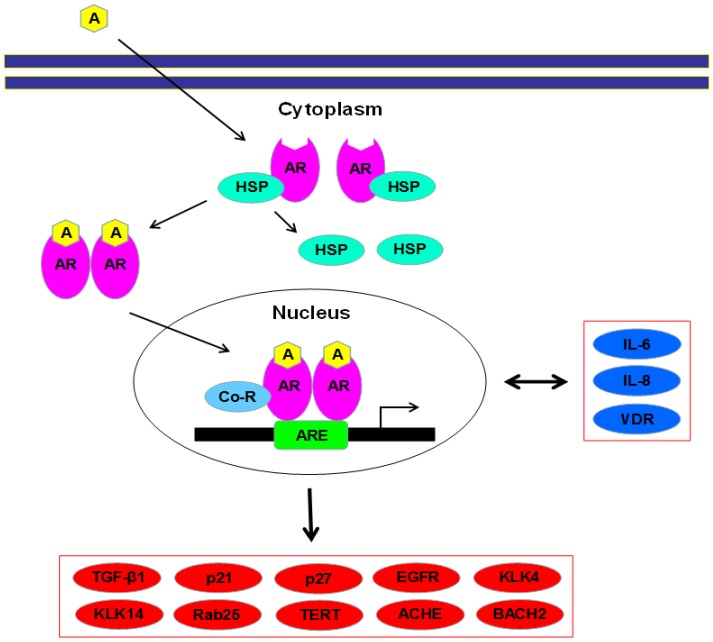
Androgen receptor (AR) and related signals in ovarian cancer cells. AR, as an inactive form, locates in the cytoplasm coupling with heat shock proteins. Upon binding of androgens, AR is released from them, forms a homodimer, and is translocated into the nucleus where it, along with co-regulators, binds to an androgen response element, leading to transcriptional regulation of various target genes listed (red and blue molecules). In particular, IL-6, IL-8, and VDR (blue) have been shown to serve as not only downstream effectors of AR but also its upstream regulators in ovarian cancer cells. A = androgen; ACHE = acetylcholinesterase; AR = androgen receptor; ARE = androgen response element; BACH2 = basic leucine zipper transcription factor 2; Co-R = co-regulator; EGFR = epidermal growth factor receptor; HS*P* = heat shock protein; IL = interleukin; KLK = kallikrein; TERT = telomerase reverse transcriptase; TGF-β1 = transforming growth factor-β1; VDR = vitamin D receptor.

Emerging evidence has suggested the role of androgen-mediated AR activation in the outgrowth of various types of malignancies, including ovarian cancer. Figure 1 illustrates the current knowledge on the activation of AR and related signaling pathways, including upstream regulators and downstream effectors, in ovarian cancer cells. In this section, we describe epidemiological/clinical and preclinical observations indicating the potential involvement of AR signaling in ovarian cancer progression.

### 3.1. Androgen Levels and Ovarian Cancer Relapse Risk

The impact of preoperative serum levels of androgens in women with ovarian cancer on the prognosis has been assessed. In 35 patients with epithelial malignancy of the ovary, low levels of DHEA-S (≤1300 nmol/L) were found to associate with a significantly shorter survival (*P* = 0.001), but those of total testosterone (≤0.016 nmol/L; *P* = 0.15) or androstenedione (≤3.9 nmol/L; *P* = 0.13) were not in a univariate setting [53]. However, multivariate analysis revealed testosterone (hazard ratio (HR) = 0.29, 95% CI = 0.08–1.02, *P* = 0.02) (as well as progesterone and the presence residual tumor), but not DHEA-S (*P* = 0.88) (as well as FIGO stage and histological tumor grade), as an independent prognosticator [53]. Another study in 52 cases of ovarian cancer showed that none of 34 patients who had disease relapse had elevated serum levels of testosterone (>1 ng/mL) preoperatively [54]. Nonetheless, in these 34 patients, testosterone levels at the time of initial treatment were significantly higher than those at relapse [54]. Thus, there are inconsistent findings on the usefulness of measurement of androgen levels in predicting postoperative disease progression in patients with ovarian cancer.

### 3.2. AR Expression in Ovarian Cancer and Its Prognostic Significance

PCR-based methods have detected *AR* gene expression in some primary cell cultures derived from human ovarian cancer and established human ovarian cancer cell lines, such as OVCA3 [23,29,55]. Then, western blotting has confirmed AR protein expression in these ovarian cancer primary cultures or cell lines [29,55]. In addition, binding of DHT to the AR with high affinity and low capacity was documented in not only OVCA3 but also other human ovarian cancer cell lines, including SKOV3 and HEY [29].

In surgical specimens, earlier studies using ligand binding assays indicated AR positivity in 85 (90.4%) of 94 [56] and 75 (91.5%) of 82 [57] ovarian tumors. Western blotting analysis in serous carcinoma tissues also detected AR signals in 69% of primary tumors (n = 49) versus 45% of metastatic tumors (n = 11) [28]. Four immunohistochemical studies in ovarian tissues published in 2000 or before demonstrated AR expression in 46% of carcinomas [58], 100% of benign tumors and 82% of malignant tumors [59], 64% of carcinomas plus one case of granulosa cell tumor [26], and 27% of carcinomas plus two cases of borderline tumor [60], respectively.

Table 1 summarizes the findings in more recent immunohistochemical studies [61,62,63,64,65,66] assessing the expression of AR in ovarian cancers and its associations with tumor grade/stage and/or patient outcomes. In these studies, the rates of AR positivity ranged from 10% to 68%. The positive rate of AR expression in serous carcinomas (23%) was found to be significantly higher than that in non-serous carcinomas (8%) in one of the studies [63], while others showed no significant differences in AR positivity among histological subtypes of carcinoma [61,62,65]. In addition, two of four studies involving different grades of tumors showed significant down-regulation of AR expression in higher grade tumors [62,65]. However, there were no significant associations between AR expression and tumor stage [61,62,63,64,65]. Prognostic significance of AR expression has also been determined in these studies. In three of them [62,64,65], AR positivity was shown to correlate with significantly better outcomes. Interestingly, other studies [61,63,66] showed opposite results (i.e., HR > 1), although they were not statistically significant. 

Thus, AR expression particularly in ovarian serous carcinomas is likely associated with less aggressive behavior. However, there appear to be limited amounts of non-serous and/or low grade cases examined. Further studies involving these cases are therefore required to determine the status of AR expression in various subtypes of ovarian tumors and its prognostic impact in both low and high grade/stage tumors.

### 3.3. AR CAG Repeat Polymorphisms and Prognosis of Ovarian Cancer

As aforementioned, the length of AR CAG repeats is inversely associated with its transcriptional activity even in ovarian cancer cells [32]. Shorter CAG repeat lengths in ovarian cancer cells are also associated with induction of their proliferation in the presence or absence of androgen [32] as well as S-phase cell cycle population [55].

As shown in men with prostate cancer [67], ovarian cancer patients with short (≤19) CAG repeats had significantly higher risks of disease recurrence (progression-free survival (PFS): 5.5 versus 19.4 months, *P <* 0.0001) and death (overall survival (OS): 9 versus 32.6 months, *P* = 0.0007) [33]. Associations between short CAG repeats and worse outcomes were also seen in subgroups of patients with or without other risk factors for ovarian cancer, including obesity (BMI ≥25 or <25) [68] or *BRCA2* mutation [69]. Another group also demonstrated that longer (≥23) CAG repeats are associated with better disease-free survival in all 69 ovarian cancer patients (HR = 0.45, 95% CI = 0.22–0.93, *P* = 0.031) or better disease-free survival (HR = 0.17, 95% CI = 0.05–0.55, *P* = 0.003)/OS (HR = 0.39, 95% CI = 0.17–0.91, *P* = 0.030) in 35 patients with tumor exhibiting TP53 protein overexpression [38]. Thus, AR activity appears to positively correlate with the progression of ovarian cancer. However, in a cohort of >300 ovarian cancer patients, no strong associations between CAG repeats (≤21 versus ≥22) and survival were observed [70].

### 3.4. Androgen-Mediated AR Signaling and the Ability of Ovarian Cancer Cell Proliferation/Invasion

The effects of androgen treatment on the cell growth of AR-positive ovarian cancer have been assessed in a substantial number of studies. First, mibolerone increased DNA synthesis in two established cell lines as well as in 5 of 9 primary cultures examined [24]. Second, DHT (e.g., 10–100 nM) considerably induced cell proliferation of established AR-positive lines [26,47,71,72,73,74], and anti-androgens, including flutamide/hydroxyflutamide and bicalutamide, blocked the stimulatory effects of DHT [47,72,73,75]. Other androgens, such as testosterone [47], androstenedione [74], and a synthetic androgen methyltrienolone [76], were also shown to increase the proliferation of cancer cells. Third, DHT increased S-phase population in a primary culture [55] or OVCAR3 [73]. Fourth, DHT blocked TGF-β1-induced growth inhibition in OVCAR3 and SKOV3 cells [29,71]. In addition, western blotting showed an increase in AR expression by DHT treatment in OVCAR3 cells [26].

Animal models have also been used to investigate the effects of androgens on the progression of ovarian cancer. In mouse xenograft models, DHT treatment resulted in significant induction of tumor growth [77,78], and an AR inhibitor enzalutamide antagonized the DHT effects [78]. Similarly, in ovarian cancer xenograft-bearing male mice, orchiectomy was shown to considerably reduce tumor growth [77,79].

It was additionally shown that DHT induced cell motility and invasion of an ovarian cancer line [79]. Methyltrienolone [76] and another AR ligand, medroxyprogesterone [80], also increased the ability of cell invasion of AR-positive ovarian cancer lines, although the latter is classified as a progestin. All of these studies have thus demonstrated in vitro and in vivo data indicating that androgens promote cell proliferation/invasion of ovarian cancer via the AR pathway. Nonetheless, conflicting findings have been documented in two studies that show significant inhibition of the cell viability of 25 primary cultures established from various subtypes of ovarian carcinomas as well as borderline and benign tumors by 0.1 pM–100 nM testosterone [81] and marginal inhibition of ovarian cancer cell invasion by 1 µM DHT [80].

### 3.5. Upstream Regulators of AR Signaling in Ovarian Cancer Cells

The molecules/pathways that regulate AR signaling in ovarian cells have not been well characterized. Interleukin (IL)-6 and IL-8 are cytokines that are implicated in, for example, cell proliferation/invasion and angiogenesis in various types of malignancies. Indeed, in patients with ovarian carcinoma, elevated levels of serum IL-6 and IL-8 were associated with significantly poor prognosis/chemoresistance [82]. In SKOV3 cells cultured in the absence of androgens, IL-6 and IL-8 were shown to induce their proliferation, which was completely blocked by specific neutralizing antibodies for IL-6/IL-8, as well as to up-regulate the expression and transcriptional activity of AR, which was not blocked by flutamide [72]. Moreover, IL-6- and IL-8-enhanced AR transactivation was blocked by pretreatment with SB202190 (p38 MAPK inhibitor)/PD98059 (MEK1/2 inhibitor)/AG879 (ERRB2 inhibitor) and PP2 (Src inhibitor), respectively. Meanwhile, DHT was found to increase IL-6 and IL-8 secretion from SKOV3 cells, which was antagonized by flutamide. These findings suggest that cytokines induce the growth of ovarian cancer cells at least partially via activation of AR. Additionally, in primary cultures of human non-neoplastic ovarian surface epithelial cells, IL-4 was shown to reduce the expression levels of AR mRNA and protein [83], which was not seen when SB203580 (p38 MAPK inhibitor) was pretreated [84].

### 3.6. Downstream Effectors of AR Signaling in Ovarian Cancer Cells

As described above, DHT likely induces the activity of IL-6 and IL-8 in SKOV3 cells, while IL-6 and IL-8 activated the AR pathway [72]. These observations suggest that androgen-mediated AR signaling and IL-6/IL-8 modulate reciprocally in ovarian cancer cells.

Transforming growth factor β1 (TGFβ1) is a polypeptide member of the TGFβ superfamily that controls a variety of cellular processes, including cell proliferation and differentiation, via binding to specific receptors (e.g., TGFβR1, TGFβR2) typically followed by activation of a cell cycle inhibitor p21 as well as smad2/3 [85]. Immunohistochemistry showed significant down-regulation of TGFβR1 expression in ovarian carcinoma tissues, compared with normal ovaries [27]. In addition, TGFβ1 did not significantly inhibit the cell proliferation in 18 (78%) of 23 ovarian cancer primary cultures [86], indicating that TGFβ1 signaling might be impaired in ovarian cancer cells. Then, incubation with DHT in ovarian cancer cell lines was found to down-regulate the expression of TGFβR1/TGFβR2 at mRNA [29] and protein [27] levels as well as that of p21 protein [27], resulting in blockade of TGFβ-mediated growth inhibition [71].

p27^Kip1^ is an enzyme inhibitor that plays a key role in controlling the progression of cell cycle at the G1 phase, as well as other cellular processes such as apoptosis, cell motility, and autophagy. In OVCAR3 cells, DHT was shown to decrease/increase the G1/S phases, respectively, down-regulate the expression of p27, and induce its degradation independent of phosphorylation status [87]. Interestingly, flutamide did not prevent DHT-induced p27 degradation, and other androgens, including testosterone and DHEA, failed to affect p27 expression/degradation [87], suggesting that DHT modulates the cell cycle progression of ovarian cancer not via the traditional AR pathway. Meanwhile, DHT induced direct binding of p27 to S-phase kinase-associated protein 2 in OVCAR3 cells [87].

Epidermal growth factor receptor (EGFR) is a member of the ErbB family of proteins and is known to involve the progression of various types of malignancies through downstream signaling pathways including MAPK. The expression of EGFR was detected in the majority of serous adenocarcinomas of the ovary (i.e., 48 of 49 primary tumors; 11 of 11 metastatic tumors) and was also positively correlated with that of AR (i.e., r = 0.49, *P <* 0.001) [28]. By contrast, AR overexpression (CAG repeat length = 21) in SKOV3 cells was associated with the increased expression of a phosphorylated form of EGFR [32]. Increases in AR CAG repeats also resulted in considerable reduction in the expression of phospho-p44/p42 MAPK [32]. 

Kallikreins (KLKs; KLK1–KLK15) are a large family of serine proteases that physiologically function as, for example, regulators of homeostasis. In breast [88] and prostate [89] cancers, KLK4 has been shown to be overexpressed and further up-regulated by androgen treatment. A putative androgen response element (ARE) has also been identified within the promoter of *KLK4* gene [90]. A PCR-based analysis revealed that *KLK4* was positive in 69 (55%) of 147 ovarian cancer tissue specimens, which was strongly associated with higher tumor grade or stage as well as poorer prognosis [91]. Up-regulation of KLK14 expression by DHT has also been demonstrated and an anti-androgen nilutamide at least partially blocked the DHT effect [92]. However, this study [92] using a quantitative PCR method showed that *KLK14* gene expression was down-regulated in ovarian cancer specimens, compared with normal ovary or benign ovarian tumor tissues, and that its positivity in cancers was associated with better rates of PFS (HR = 0.53, 95% CI = 0.31–0.93, *P* = 0.027) and OS (HR = 0.42, 95% CI = 0.21–0.84, *P* = 0.014) even in a multivariate setting as well as enhanced sensitivity to chemotherapy.

Rab25, a small GTPase, has been considered as an oncogene in several types of malignancies, including ovarian cancer, via inducing cell proliferation and suppressing apoptosis/anoikis [93]. The levels of *Rab25* gene expression were significantly higher in stages III/IV ovarian cancers than in stages I/II tumors or normal ovary tissues, and elevated *Rab25* expression was associated with a significantly worse survival rate in patients with ovarian cancer [93]. A significant positive correlation between Rab25 and AR expression was also found in ovarian cancer specimens [73], although there is no direct evidence indicating that androgens regulate the expression/activity of Rab25.

1,25-dihydroxyvitamin D_3_, via binding to another member of the steroid hormone receptor superfamily, vitamin D receptor (VDR), plays a critical role in regulating the growth and differentiation of a variety of organs, including the ovary. Indeed, treatment with an active metabolite of vitamin D, 1,25-dihydroxyvitamin D_3_, in OVCAR3 cells significantly induced their proliferation [26]. In addition, immunohistochemistry in surgical specimens detected VDR in 6 (43%) of 14 ovarian tumors [26]. More importantly, in OVCAR3 cells, DHT increased nuclear expression of VDR, while 1,25-dihydroxyvitamin D_3_ increased that of AR [26]. These findings suggest a cross-talk between AR and VDR signals in ovarian cancer cells.

Telomerase reverse transcriptase (TERT) is an essential enzyme for regulating cell immortalization as well as long-term tumor growth. TERT is activated in the majority of malignancies, including prostate cancer in an androgen-dependent manner [94] as well as ovarian cancer [95]. In OVCAR3 cells, both testosterone and androstenedione were shown to induce the expression of *TERT* gene as well as the activity and phosphorylation of telomerase, which was prevented by pretreatment with a phosphatidylinositol-3-kinase inhibitor [74].

DNA microarray analysis in morphologically normal ovarian surface epithelial cells from women with germline mutations of *BRCA1* or *BRCA2* and ovarian cancer cells identified genes that were up-regulated by DHT treatment [96]. These genes included *acetylcholinesterase* (*ACHE*) and *basic leucine zipper transcription factor 2* (*BACH2*) whose functions in ovarian cancer remain poorly understood. The expression of ACHE and BACH2 proteins was also found to be elevated in ovarian cancer tissue specimens, compared with benign tumors or normal ovaries, and associate with worse patient outcomes [96].

### 3.7. Co-Regulators That Modulate AR Transcriptional Activity in Ovarian Cancer

As seen in other types of cells, androgens activate ARE-mediated transcription in ovarian cancer cells [32]. Similarly, androgens increase the expression levels of AR and facilitate its nuclear translocation in ovarian cancer cells [97]. Meanwhile, a variety of co-regulators, comprising co-activators and co-repressors that physically interact with steroid hormone receptors, are known to modulate AR activity [98], although the majority of these interact with other receptors as well. Of these AR co-regulators, several have indeed been assessed in ovarian cancer tissue specimens and/or cell lines, although their functional role in AR activity or tumor growth remains largely unknown in ovarian cancer. Nonetheless, the findings in AR co-regulators suggest that AR activation is associated with the promotion of ovarian tumorigenesis and tumor growth.

Nuclear receptor coactivator 3 (NCOA3), also known as AIB1, SRC-3, or TRAM-1, was cloned as a gene which was amplified in breast cancer [99]. It belongs to the SRC-1 family and can physically interact with AR as a co-activator [100]. In BG-1 ovarian cancer cells, NCOA3 was found to be amplified, whereas SRC-1 and TIF2 (SRC-2) were not [99]. Amplification of the *AIB1* locus (i.e., 20q12) was also detected in 25% of sporadic ovarian carcinomas [101]. In addition, immunohistochemistry in ovarian carcinoma tissue samples revealed that strong NCOA3 expression was associated with higher stages (i.e., stages III and IV) and worse patient outcomes (HR = 1.349, *P* = 0.015 in a multivariate analysis for OS) [102]. Patients with shorter (≤28) CAG repeats in the *NCOA3* gene were also shown to have a higher risk of disease progression (HR = 1.28, 95% CI = 1.01–1.66, *P* = 0.05 in a multivariate analysis for overall survival) after initial cytoreductive surgery for ovarian cancer [103]. By contrast, in primary cultures derived from ovarian cancer, DHT was shown to reduce the expression levels of *NCOA3* (in 5 of 8 primary cultures; up-regulated in 2 primary cultures) as well as *SRC-1* (in 3 of 8 primary cultures; no significant changes in others), while the levels of *NCOA3* (but not *SRC-1*) were approximately 2-fold higher in ovarian cancers than in primary cultures of ovarian surface epithelium [71]. 

ARA70 is a 70 kDa co-regulatory protein that preferentially activates the AR upon androgen binding, although its interactions with other steroid hormone receptors have been documented [104,105]. In situ hybridization identified high levels of *ARA70* mRNA in 17 of 20 ovarian carcinomas, while ARA70 was negative or faintly positive in ovarian surface epithelium (versus moderately positive in theca cells and weakly positive in granulosa and stromal cells) [106]. In a study described above [71], the levels of *ARA70* expression were approximately 2-fold higher in primary cultures derived from ovarian cancer than in those of ovarian surface epithelium. Moreover, in these primary cultures of ovarian cancer, both up-regulation (5 of 8) and down-regulation (3 of 8) of *ARA70* expression by DHT treatment were observed [71]. 

Melanoma-associated antigen 11 (MAGEA11), originally identified in melanomas as a cancer germline antigen, has been shown to function as an AR co-activator [107]. *MAGEA11* gene expression was significantly elevated in ovarian carcinomas, compared with normal ovary tissues [108]. Moreover, in ovarian cancer, the expression of *MAGEA11* was found to correlate positively with that of other cancer germline antigen genes and inversely with DNA methylation on its promoter activity [108].

p44 is a 44 kDa AR-interacting protein originally identified as a subunit of the methylosome complex [76]. An immunohistochemical study showed that cytoplasmic p44 was more strongly expressed in normal-appearing ovarian surface epithelium, while nuclear p44 expression was predominantly observed in ovarian carcinoma [76]. Additionally, in OVCAR3 and SKOV3 cells, overexpression of nuclear p44 and silencing of p44 resulted in increases and decreases, respectively, in their proliferation and invasion in the presence of androgen [76].

## 4. Role of Androgens and AR Signaling in Chemoresistance in Ovarian Cancer

AR signaling has been linked to sensitivity to chemotherapeutic drugs in various malignancies [3], such as prostate and urothelial cancers. In a SKOV3 subline resistant to paclitaxel, AR expression was considerably up-regulated, compared with the parental cells [109,110]. Correspondingly, AR silencing in the resistant SKOV3 cells enhanced paclitaxel-mediated apoptosis and its cytotoxicity [110]. In addition, AR silencing and DHT treatment resulted in decreases and increases, respectively, in the expression of several genes, such as ABCB1, ABCB6, ABCG2, BMP5, FGFR2, and HIF0, whose silencing in SKOV3 cells also increased sensitivity to paclitaxel [110]. Thus, AR overexpression likely correlates with resistance to paclitaxel in ovarian cancer cells.

FKB506 binding protein 5 (FKBP5), also known as FKBP51, is a member of the immunophilin family and has been suggested to involve chemosensitivity presumably via functioning as a scaffold protein which recruits/regulates the expression of related genes [109,111]. In SKOV3 cells, FKBP5 was shown to form a protein complex with AR, and FKBP5 silencing resulted in increased chemosensitivity [109]. FKBP5 overexpression was also associated with increases in the expression of 6 chemoresistance-related genes described above in SKOV3 cells [109].

Several upstream regulators/downstream targets of AR and co-regulators described above have been implicated in chemoresistance in ovarian cancer. In patients with ovarian cancer, elevated levels of serum IL-6 (*P* = 0.041) and IL-8 (*P* = 0.041) were associated with poorer initial response to paclitaxel [82]. Significantly better response to platinum-based chemotherapy was seen in patients with *KLK14*-positive ovarian cancer [67 of 70 (95.7%)], compared to those with *KLK14*-negative tumor [58 of 72 (77.3%)] [92]. Low expression of NCOA3/SRC3 protein in ovarian cancer tissues, as compared with high expression, was associated with better prognosis in patients undergoing carboplatin monotherapy (*P <* 0.001), but not in those undergoing combined carboplatin and paclitaxel therapy (*P* > 0.05) [102].

## 5. Current Evidence from Clinical Trials of Androgen Deprivation Therapy in Patients with Ovarian Cancer

As aforementioned, preclinical evidence has suggested that AR activity correlates with the progression of ovarian cancer, while conflicting findings exist. Several clinical studies have then been conducted to assess the efficacy of anti-androgens with or without gonadotropin releasing hormone agonists in patients with ovarian cancer. Of note, androgen deprivation therapy with anti-androgens, such as flutamide and bicalutamide, and/or gonadotropin releasing hormone agonists that eventually decrease androgen secretion via suppressing follicle stimulating hormone release form the pituitary grand, such as goserelin, have been widely used in men with prostate cancer without severe adverse effects [2].

In a case series [112], flutamide (750 mg/day) was orally given to 12 patients with ovarian carcinoma showing progression after standard treatment. One patient achieved stabilization of disease for 8 months and others still had progressive disease, although no unacceptable adverse effects of flutamide were seen.

At least 3 phase II trials have been conducted and completed. First, 68 patients who had recurrence with progressive disease after platinum-based chemotherapy were enrolled, and 32 of them completed oral flutamide treatment (750 mg/day) for at least 2 months [113]. Of the 32 patients, 2 (6.3%) showed considerable effects (1 complete response lasted for 44 weeks and 1 partial response lasted for 72 weeks), and 9 (28.1%) had stable disease for a median of 24 weeks (range: 12–48). Second, 24 patients with stage III or IV ovarian carcinoma who had measurable disease following chemotherapy received flutamide (300 mg/day) [114]. Partial response and disease stabilization were observed in one case (4.3% of 23 evaluable patients) lasted for 3 months and two cases (8.7%) lasted for 7–8 months, respectively. Third, ovarian cancer patients who underwent surgical cytoreduction and platinum-based chemotherapy, failed the primary regimen, and subsequently achieved complete response (i.e., CA125 ≤35 U/mL, no evidence of disease on computed tomography scans) to the second (n = 21) or third/fourth (n = 11) regimen were treated with oral bicalutamide (30 mg/day) and subcutaneous goserelin (3.6 mg/4 weeks) [115]. PFSs were 11.4 months (95% CI = 10.2–12.6) in those with second disease remission and 11.9 months (95% CI = 10.8–14.1) in those with third or fourth disease remission, which were even shorter than the predetermined value of 16.5 months the investigators considered the approach worthy of further assessment. In addition, although AR activity might have a predictive value for responses to androgen deprivation therapy, AR protein expression or CAG repeat length was not significantly associated with PFS in these patients [115].

Current evidence from limited clinical trials thus suggests that only a subset of patients with ovarian cancer benefit from androgen deprivation therapy consisting of 1st generation non-steroidal anti-androgenic drugs, while their side effects are generally tolerable. Accordingly, further studies are required to determine actual benefits of androgen deprivation therapy and its optimal regimens as well as to select appropriate candidates via, for example, AR expression, AR polymorphism/splice variants, and activity of AR downstream targets.

Enzalutamide is an oral AR signaling inhibitor which not only blocks androgen binding to AR but also prevents AR nuclear translocation, DNA binding, and co-activator recruitment [116]. It was approved by the US Food and Drug Administration for the treatment of castration-resistant prostate cancer in 2012. Recently, a phase II study is being conducted to assess the efficacy of enzalutamide in women with AR-positive ovarian cancer (NCT 01974765) [117].

## 6. Conclusions

AR expression has been confirmed in both normal ovarian tissues and ovarian carcinomas. Clinical or epidemiological studies have revealed the relationship between the risk of ovarian cancer and the level of serum androgens, treatment with androgenic/anti-androgenic drugs, or AR CAG repeat polymorphisms. Preclinical experiments have further suggested that AR activation correlates with the induction of ovarian tumorigenesis and cancer progression as well as chemoresistance in ovarian cancer. Accordingly, AR inactivation has the potential of being not only a promising chemopreventive and/or therapeutic approach against ovarian cancer but also a means of chemosensitization, particularly in patients with AR-positive tumor. Indeed, androgen deprivation therapy with anti-androgens and/or gonadotropin releasing hormone agonists has often been used in, for example, men with prostate cancer and may therefore be able to be readily applied to female patients. Remarkably, conflicting findings exist and demonstrate opposite or minimal effects of androgen-mediated AR signals on ovarian cancer outgrowth. Further research is thus required to precisely determine the functional role of AR signaling in ovarian cancer.

## Figures and Tables

**Table 1:** Immunohistochemical studies assessing AR expression in ovarian cancer tissue specimens.

Author, Year [Reference]	n	AR Positivity	Histological Subtype of Carcinoma						Tumor Grade				FIGO Stage					PFS		OS	
			Serous	EM	CC	MUC	Others	*p*	G1	G2	G3	*p*	I	II	III	IV	*p*	HR (95%CI) (ref: AR-)	*p*	HR (95%CI) (ref: AR-)	*p*
Lee, 2005 [61]	286	44%	48% (104/217)	38% (11/29)	20% (3/15)	17% (1/6)	32% (6/19)	0.16	46% (6/13)	36% (8/22)	44% (111/251)	0.76	32% (10/31)	32% (7/22)	48% (87/180)	40% (21/53)	0.18		NA	1.1 (0.8–1.5)	0.53
Nodin, 2010 [62]	154	18%	21% (19/90)	20% (7/35)	7% (2/29)			0.21	21% (10/47) 33% (7/21)		17% (18/107) 15% (10/67)	<0.01	15% (4/26) 33% (2/6)	11% (2/18) 29% (2/7)	24% (18/75) 24% (13/54)	14% (3/22) 8% (1/14)	0.67 0.14		NA NA	NA 0.5 (0.3–1.0)	0.54 0.04
de Toledo, 2014 [63]	152	15%	23% (17/75)	8% (6/77)				0.01	16% (6/37)	16% (17/109)		0.20	14% (8/56)	16% (15/96)			0.99	1.8 (0.5–6.6)	0.37	1.9 (0.6–58)	0.27
Martins, 2014 [64]	216	68%	68% (147/216)					NA				NA	71% (29/41)	75% (18/24)	66% (80/121)	63% (15/24)	NS		NA	0.6 (0.4–0.9)	0.01
Jönsson, 2015 [65]	118	44%	45% (39/87)	42% (13/31)				0.80	77% (13/17)	42% (10/24)	38% (23/60)	0.03	73% (11/15)	31% (5/16)	44% (31/70)	29% (5/17)	0.09	0.5 (0.3–0.8)	<0.01	0.4 (0.2–0.6)	<0.01
van Kruchten, 2015 [66]	121	10%	11% (9/85)	19% (4/21)	0% (0/11)	0% (0/4)		NS				NA					NA	1.3 (0.6–2.6)	0.53	1.8 (0.9–3.7)	0.10

AR: androgen receptor; CC: clear cell; CI: confidence interval; EM: endometrioid; HR: hazard ratio; MUC: mucinous; NA: not assessed; NS: not significant; OS: overall survival; PFS: progression-free survival.
